# *Lactiplantibacillus plantarum*, Duodenal Hydroxyphenyllactic Acid and Iron: Insights from a Rat Model of a High-Fat Iron-Deficient Diet

**DOI:** 10.3390/nu17213454

**Published:** 2025-11-01

**Authors:** Katarzyna Skrypnik, Agnieszka Olejnik-Schmidt, Marcin Schmidt, Damla Selvan, Joanna Suliburska

**Affiliations:** 1 Department of Human Nutrition and Dietetics, Poznan University of Life Sciences, Wojska Polskiego St. 31, 60-624 Poznan, Polandjoanna.suliburska@up.poznan.pl (J.S.); 2 Department of Food Biotechnology and Microbiology, Poznan University of Life Sciences, Wojska Polskiego St. 48, 60-627 Poznan, Poland; agnieszka.olejnik-schmidt@up.poznan.pl (A.O.-S.); marcin.schmidt@up.poznan.pl (M.S.)

**Keywords:** probiotics, duodenum, high-fat diet, iron deficient diet, hydroxyphenyllactic acid, HPLA

## Abstract

**Background**: *Lactiplantibacillus plantarum* synthesizes in vitro hydroxyphenyllactic acid (HPLA)—an iron-reducing agent supposed to facilitate duodenal Fe absorption. So far, no such in vivo HPLA production has been established. This study aimed to investigate the ability of *Lactiplantibacillus plantarum* to produce HPLA in the duodenum in rats on a high-fat iron-deficient diet. **Methods**: Rats were fed a high fat (HF) diet; HF, Fe-deficient diet (HFDEF); or control (C) diet for 8 weeks. Over the next 8 weeks, animals in the C and HF groups continued on their respective diets, while animals in the HFDEF group were divided into six subgroups and received combinations of an HF, Fe-deficient diet with *Lactiplantibacillus plantarum* (Lp), *Latilactobacillus curvatus* (Lc), and Fe supplementation (HFDEF, HFDEFFe, HFDEFLp, HFDEFLc, HFDEFFeLp, and HFDEFFeLc). Duodenal and faecal samples were collected. **Results**: No significant differences were observed in HPLA content in the duodenum and faeces, nor in Fe chelating abilities in faeces, between study groups at the completion of the study. Fe content in faeces was higher in the HFDEFFe group than in the C, HF, HFDEF, HFDEFLp, and HFDEFLc groups. Fe content in faeces was higher in the HFDEFFeLp and HFDEFFeLc groups than in the HFDEF and HFDEFLc groups. **Conclusions**: *Lactiplantibacillus plantarum*, whether alone or with oral Fe, does not influence duodenal and faecal HPLA content, nor does it affect faecal Fe chelating abilities in rats on the HF, Fe-deficient diet.

## 1. Introduction

Currently, one of the most emerging nutritional concerns worldwide is dietary iron (Fe) deficit. It is estimated that globally, more than 16,430 per 100,000 people are affected by inadequate dietary iron intake, which results in 423.7 per 100,000 DALYs (disability-adjusted life years) [1]. Moreover, contemporary dietary patterns are characterised by high fat consumption [2,3,4,5,6], which is associated with numerous detrimental health states, including obesity, type 2 diabetes, arterial hypertension, lipid disorders, and cardiovascular diseases [7,8].

**Figure 1:**
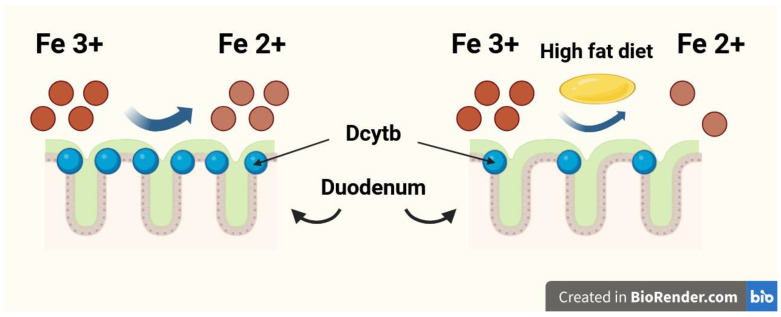
The iron (Fe) reduction process in the duodenum by duodenal cytochrome b (Dcytb).

The duodenum is the organ most responsible for dietary Fe absorption, where dietary ferric Fe (Fe^3+^) is reduced to the ferrous form (Fe^2+^) by the membrane-bound ferrireductase—duodenal cytochrome b (Dcytb). Subsequently, Fe^2+^ enters the duodenal enterocytes through the divalent metal transporter 1 (DMT1) [9,10]. A high-fat diet may impair dietary Fe absorption in the duodenum by reducing Dcytb expression [11,12]. The Fe reduction process in the duodenum by Dcytb is shown in Figure 1. The gut microbiota plays a significant role in duodenal Fe absorption. Intestinal bacteria convert Fe into Fe^2+^ [13], reduce the quantity of substances with high affinity for Fe, such as ellagic acid [14], and can even regulate the expression of Fe-related genes in an epigenetic manner, including those encoding DMT1 and Dcytb [15]. Multiple studies recently meta-analysed by Vonderheid et al. unequivocally confirmed that probiotics are also able to improve gut absorption of dietary Fe [16].

**Figure 2:**
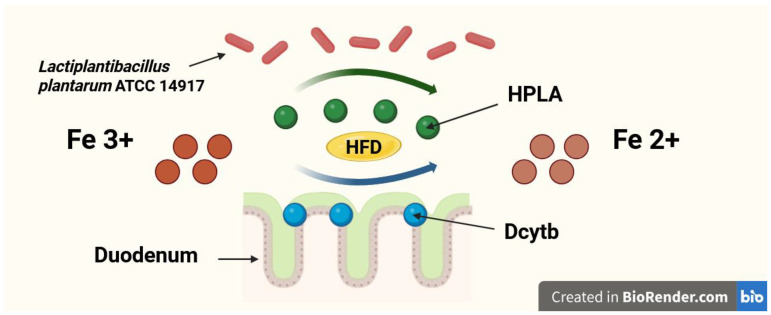
The possible mechanism of iron (Fe) reduction in duodenum by hydroxyphenyllactic acid (HPLA) and duodenal cytochrome b (Dcytb) in conditions of a high-fat diet (HFD).

A newly discovered and still underexplored mechanism by which probiotics may enhance dietary Fe absorption is the production of *p*-hydroxyphenyllactic acid (HPLA). HPLA is a ferric-reducing factor that facilitates the reduction of Fe^3+^ to Fe^2+^, promoting its absorption in the duodenum [17,18]. However, the association between dietary iron and HPLA synthesized by probiotic bacteria was only explored in in vitro experiments [17,18]. Still, studies investigating the ability of probiotics to produce HPLA in in vivo models, especially in the duodenum—where its influence on dietary Fe absorption is supposed to be the most significant—are lacking. Furthermore, no such studies were performed in conditions of high-fat Fe-deficient diet. The possible mechanism of Fe reduction in duodenum by HPLA and Dcytb in conditions of a high-fat diet is shown in Figure 2. On the other hand, probiotics are capable of chelating Fe [19] and by this mechanism may also reduce Fe absorption in the gut [20,21].

HPLA is profusely synthesised by *Lactiplantibacillus plantarum* (formerly: *Lactobacillus plantarum*) [17,18]. Moreover, the probiotic strain *Lactiplantibacillus plantarum* ATCC 14917 does not absorb Fe for its own growth and, as a result, does not reduce the quantity of Fe available for absorption in the duodenum [22]. By contrast, *Latilactobacillus curvatus* (formerly: *Lactobacillus curvatus*) is a probiotic strain that produces HPLA in only negligible amounts [18]. Thus, *Latilactobacillus curvatus* provides a useful comparison for investigating whether the effects observed in scientific studies are due to HPLA production or probiotic supplementation itself.

In our study we hypothesise that in conditions of a high-fat iron-deficient diet, probiotic bacteria *Lactiplantibacillus plantarum* ATCC 14917 produces HPLA in the duodenum. In our previous studies on such dietary conditions, we demonstrated that *Lactiplantibacillus plantarum* is able to exert a significant influence on Fe [23,24,25]. In this study, we further investigated the selected potential mechanisms underlying the effects observed so far.

The aim of this study was to investigate the potential ability of probiotic bacteria *Lactiplantibacillus plantarum* ATCC 14917 to produce hydroxyphenyllactic acid in the duodenum in conditions of a high-fat iron-deficient diet. Additionally, we investigated HPLA and Fe content in the faeces and Fe chelation abilities of *Lactiplantibacillus plantarum* ATCC 14917 and *Latilactobacillus curvatus* in the faeces. The selection of probiotic strains and the determination of HPLA content in an in vivo rather than in vitro model constitute the uniqueness of this study.

## 2. Materials and Methods

### 2.1. Study Design

This study was conducted on 8-week-old female Wistar rats from the same breeding stock (*n* = 64). Before the onset of the study, the rats’ body masses were recorded. The experiment was carried out in two stages. In the first stage (8 weeks), the animals were randomly assigned to three groups using a random number generator. Each group was fed a different diet: the C group (*n* = 8, control group) was fed a standard diet, the HF group (*n* = 8) was fed a high-fat diet, and the HFDEF group (*n* = 48) was fed a high-fat, Fe-deficient diet. At the end of this stage, the body masses of all animals were recorded again.

In the second stage (8 weeks) of the experiment, animals in the C and HF groups continued on their respective standard and high-fat diets. The HFDEF group was further divided into six subgroups of eight rats each using a random number generator: HFDEF, HFDEFFe, HFDEFLp, HFDEFLc, HFDEFFeLp, and HFDEFFeLc.

- HFDEF subgroup: Continued on the high-fat, Fe-deficient diet from the first stage.
- HFDEFFe subgroup: Fed a high-fat, Fe-deficient diet supplemented with Fe in the form of Fe gluconate (120 mg Fe/kg diet).
- HFDEFLp subgroup: Fed a high-fat, Fe-deficient diet with the probiotic *Lactiplantibacillus plantarum* (daily dose: 5 × 10^9^ colony-forming units [CFU]).
- HFDEFLc subgroup: Fed a high-fat, Fe-deficient diet with the probiotic *Latilactobacillus curvatus* (daily dose: 5 × 10^9^ CFU).
- HFDEFFeLp subgroup: Fed a high-fat, Fe-deficient diet with *Lactiplantibacillus plantarum* (daily dose: 5 × 10^9^ CFU) and Fe supplementation (Fe gluconate; 120 mg Fe/kg diet).
- HFDEFFeLc subgroup: Fed a high-fat, Fe-deficient diet with *Latilactobacillus curvatus* (daily dose: 5 × 10^9^ CFU) and Fe supplementation (Fe gluconate; 120 mg Fe/kg diet).

Upon completion of the second stage of the experiment, all rats were weighed. Whole-day faeces collection was conducted over the last 3 consecutive days. All rats were subsequently euthanised by carbon dioxide asphyxiation, preceded by a 12 h fasting period. Euthanasia was performed in the morning, ensuring all animals were sacrificed within the same time period. Immediately after euthanasia, dissections were performed. During the procedure, duodenal samples were collected.

Eight rats per study subgroup is considered a standard and sufficient quantity to achieve high enough statistical power to detect a significant effect of an intervention. The statistical power for detecting significant differences between the study groups was estimated based on the HPLA content in the duodenum. A sample size of eight rats per group was determined to provide a statistical power of approximately 83% at the α level of 0.05. Moreover, increasing the number of rats in the experimental subgroups was not permitted because of ethical considerations [26,27]. The Fe content and HPLA levels were measured in the duodenum and faeces. Faecal microbiological analysis was also performed, and Fe chelating ability in the faeces was assessed.

**Figure 3:**
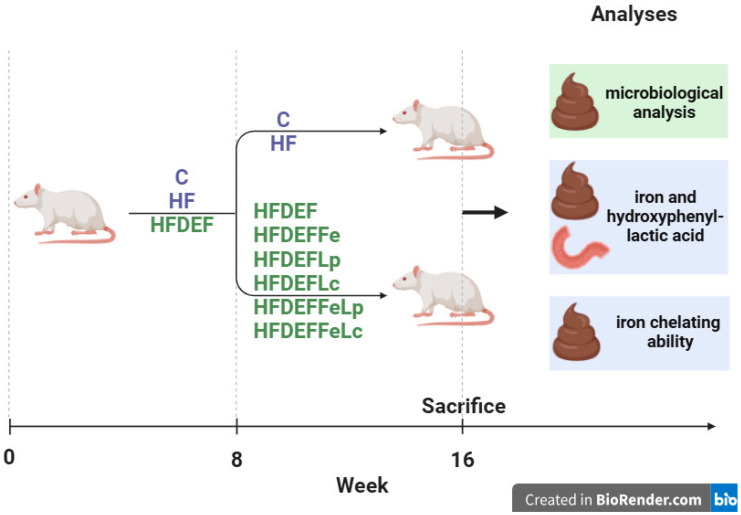
The study design. Study groups fed different diets: **C** (control group)—standard diet; **HF**—high-fat diet, **HFDEF**—high-fat iron (Fe) deficient diet; **HFDEFFe**—high-fat Fe-deficient diet supplemented with Fe; **HFDEFLp**—high-fat Fe-deficient diet with the probiotic *Lactiplantibacillus plantarum*; **HFDEFLc**—high-fat Fe-deficient diet with the probiotic *Latilactobacillus curvatus*; **HFDEFFeLp**—high-fat Fe-deficient diet with *Lactiplantibacillus plantarum* and Fe supplementation; **HFDEFFeLc**—high-fat Fe-deficient diet with *Latilactobacillus curvatus* and Fe supplementation.

The study was blinded during the results assessment phase: the personnel conducting the measurements were not informed about the rat groupings or the interventions performed. No changes were made to the methods after the study commenced. Additionally, no expected or unexpected adverse events were recorded during the experiment. This article is part of a broader research effort initiated in our previous studies [23,24,25] and advances the research by addressing issues associated with Fe content in faeces; HPLA content in duodenum and faeces; and Fe chelating ability in faeces. The study design is illustrated in Figure 3.

An 8-week period of high-fat diet feeding was chosen for the first stage of the experiment. In animal studies, alterations in body composition and fat content have been observed after 28–42 days of HF diet consumption [28]. Moreover, significantly greater increases in body weight and cardiovascular complications have been documented in numerous studies following an 8-week period of an HF diet compared with a standard diet [26,27,29].

An 8-week period (56 days) of Fe-deficient diet feeding was chosen for the first stage of the experiment. Studies have shown that daily Fe consumption at 25% of the recommended intake for 4 weeks leads to significant disturbances in whole-blood morphology and Fe content in the blood and organs of rats [30]. Additionally, changes in Fe metabolism have been observed in numerous studies on Fe-deficient feeding in rats after as little as 3–17 days [31,32].

### 2.2. Study Animals

The rats were purchased immediately before the study onset from the Greater Poland Center of Advanced Technologies (Poznań, Poland). The study was conducted in accordance with the European Communities Council Directive of 24 November 1986, Polish legal requirements, the National Institutes of Health Guide for the Care and Use of Laboratory Animals (National Institutes of Health Publication No. 80–23, Revised 1978), and protocols for animal studies approved by Poznań University of Life Sciences. The study procedures and protocol were registered and approved by the Local Bioethics Committee for Animal Studies (approval no. 57/2019). Throughout the study, the Animal Research: Reporting of In Vivo Experiments (ARRIVE) guidelines were implemented.

All procedures involving animals were conducted at the Laboratory of the Institute of Human Nutrition and Dietetics, Poznań University of Life Sciences. The animals were housed in standard, metal-free, enamel-coated stainless steel cages. They were kept under stable and controlled conditions, with two rats per cage, following a 12 h light/dark cycle. The temperature was maintained at 21 °C ± 2 °C, and the relative humidity in the animal room ranged from 55% to 65%. Veterinary supervision was provided throughout the entire study.

The rats had ad libitum access to chow and deionised water. Fresh portions of water and chow were supplied daily, and any remaining chow and water from the previous day were removed. Before the study onset, the animals underwent a 5-day acclimatisation period under the same conditions as during the experiment. During acclimatisation, the rats had unrestricted access to deionised water and an AIN-93 M diet [33] (Altromin, Lage, Germany). The baseline mean body mass of the animals after adaptation was 175.0 ± 27.1 g. Throughout the experiment, animal stress was minimised by reducing both the duration and frequency of contact between the rats and investigators, as well as by limiting the number of experimenters handling the animals during study procedures.

Female rats were selected for this study because they are more susceptible to Fe supplementation over a broader range than male rats, and alterations in Fe metabolism markers in response to Fe supply are more pronounced in females than in males [34]. Eight-week-old animals were chosen to ensure the inclusion of only mature rats. Studies on rat maturation indicate that female Wistar rats reach full maturity at approximately 41 days of age (approximately 5.9 weeks) [35].

### 2.3. Diet

The AIN-93 M diet (10.7% energy from fat [Keyes Cariogenic Diet, Rat & Mouse Diet, Diet Identification: Nutritive Composition]) was used as the standard diet [36]. To produce the high-fat (HF) diet and HF, Fe-deficient chow, a modified AIN-93 M diet was used, with lard substituted for the oil. The HF diets implemented in the experiment provided 59.3% of energy from fat [26]. Even a lower fat content (46.5% of energy intake) has been shown to significantly increase body mass and the Lee index compared with a standard diet [27]. Fe-deficient chows were produced from the AIN-93 M diet using a mixture of mineral salts lacking Fe [30]. These Fe-deficient chows contained approximately 11.8 mg Fe in the form of Fe gluconate per 1 kg of chow—roughly 25% of the recommended daily Fe intake. Typically, 40–50 mg Fe per 1 kg of diet is used to meet the Fe requirements of rats [30]. The detailed composition of the diets is presented in Supplementary Table S1 [25].

To supplement Fe, Fe gluconate in powdered form was homogenised with the diet at a dose of 120 mg Fe/kg of chow. This dose is three times higher than the standard 40–50 mg Fe/kg of chow typically used to meet the Fe requirements of study rats [30]. In previous studies on rats, this dose has been shown to influence Fe metabolism and haemoglobin levels [37]. Diet intake was monitored daily, allowing for the determination of the exact amounts of probiotics and Fe consumed by the study rats.

### 2.4. Probiotics

*Lactiplantibacillus plantarum* (American Type Culture Collection [ATCC] 14917; German Collection of Microorganisms and Cell Cultures no. DSM 20174) and *Latilactobacillus curvatus* (ATCC 25601; German Collection of Microorganisms and Cell Cultures no. DSM 20019) were obtained from DSMZ–German Collection of Microorganisms and Cell Cultures GmbH (Leibniz Institute, Braunschweig, Germany). The species purity and strain identity were ensured by DSMZ–German Collection of Microorganisms and Cell Cultures GmbH through standardized quality control procedures. Probiotic specimens in the form of a freeze-dried powder of both microorganisms were prepared in sachets, each containing 5 × 10^9^ CFU per dose. This dose was equivalent to the amount of bacteria homogenised with the diet and was administered to each rat once per day. The carrier matrix consisted of maize starch and maltodextrins. Homogenisation with the diet was chosen to avoid invasive feeding procedures, better mimic physiological conditions, and reduce the potential risk of preterm mortality in rats, thereby allowing for a smaller group size. Additionally, unlike gavage, probiotic homogenisation with the diet does not induce physiological stress. This was particularly important for the experiment because stress could have significantly influenced the study results and led to incorrect conclusions. Gavage feeding was also avoided for ethical reasons. In our previous study, probiotic homogenisation with the diet was successfully implemented, yielding significant results [38]. Alterations in Fe metabolism markers in previous studies examining the effects of probiotics on Fe metabolism have been observed after 10–40 days of probiotic administration [39,40]. Therefore, in the second stage of the experiment, the probiotic supplementation period was extended to 8 weeks (56 days). Additionally, previous experiments have demonstrated that a daily probiotic dose of 5 × 10^9^ CFU is sufficient to induce significant effects [41]; thus, the same dosage was implemented in this study.

### 2.5. Duodenum and Faeces Collection and Preparation

During dissection, the duodenum was removed, washed in saline, and weighed immediately after collection. Duodenal samples were stored at −80 °C for subsequent analyses, including HPLA and Fe content measurement. Before HPLA content determination, the duodenal samples were homogenised using an automatic homogeniser (BeadBug 6; Benchmark Scientific, Inc., Sayreville, NJ, USA).

Following the second stage of the experiment, a 24 h faeces collection was conducted over the last 3 consecutive days. Faeces were collected manually directly from the cages. The animals were housed in cages with a wire-grid bottom and a tray lined with absorbent material underneath, which prevented contact between faeces and urine. Fresh faecal pellets were carefully picked using sterile forceps immediately after defecation to minimize the risk of contamination. Only intact, dry pellets free from any visible urine traces were used for subsequent analyses. All faecal samples were handled under clean conditions, and separate sterile tools were used to avoid cross-contamination. This procedure ensured that the collected faeces were not contaminated by urine and that the samples were suitable for reliable analysis. The total faecal bacterial content, faecal Lactobacillaceae content (as described in our previous study [23]), and faecal Fe chelating ability were determined from freshly collected, non-frozen faecal samples. A portion of the faecal samples was frozen at −80 °C for subsequent analyses, including HPLA and Fe content measurement. Before HPLA content determination, the faecal samples were homogenised using an automatic homogeniser (BeadBug 6; Benchmark Scientific, Inc., Sayreville, NJ, USA).

### 2.6. Faecal Microbiological Analysis

Faecal samples were collected from all study groups before the fasting period. After collection, the sample mass was measured, and the samples were soaked in saline supplemented with 0.1% Tween 80. They were then homogenised using a stomacher device. The faecal suspension was serially diluted and plated in duplicate on Columbia Agar with 5% sheep blood (BTL, Łódź, Poland) for the enumeration of total bacteria and on MRS (De Man, Rogosa, and Sharpe) Agar (BTL, Łódź, Poland) for the enumeration of Lactobacillaceae. The plated agar media were placed in airtight boxes with anaerobic gas-generating sachets (AnaeroGen, Thermo Scientific Oxoid, Thermo Fisher Scientific, Waltham, MA, USA) and incubated at 37 °C for 72 h (Columbia Agar plates) or 48 h (MRS Agar plates). After incubation, the quantity of bacterial colonies was determined, and bacterial cell counts in faeces were calculated [23].

### 2.7. Fe and HPLA Content Determination

The Fe content in the diet was determined using flame atomic absorption spectrometry (AAS-3; Carl Zeiss, Jena, Germany) following prior digestion in 65% (*w*/*w*) spectrally pure HNO_3_ (Merck, Kenilworth, NJ, USA) using a microwave digestion system (Speedwave Xpert; Berghof, Eningen, Germany). The digested samples were then diluted with deionised water before analysis.

The mean Fe content in the diet was as follows:

- C group: 38.373 mg Fe/kg;
- HF group: 55.593 mg Fe/kg;
- HFDEF, HFDEFLp, and HFDEFLc subgroups: 27.645 mg Fe/kg;
- HFDEFFe, HFDEFFeLp, and HFDEFFeLc subgroups: 177.632 mg Fe/kg.

The accuracy of the method was verified using certified reference materials, specifically Brown Bread BCR191 (LGC Standards GmbH, Wesel, Germany) for the diet samples [23]. Although the iron-deficient diet was formulated to contain approximately 11.8 mg Fe/kg, flame atomic absorption spectrometry determination revealed slightly higher Fe content (27.645 mg Fe/kg) than expected. This discrepancy likely resulted from natural variability in raw materials and trace iron contamination during diet preparation. Nevertheless, the iron-deficient diet still provided substantially less Fe than both the control (38.373 mg Fe/kg) and iron-supplemented diets (177.632 mg Fe/kg), and thus effectively represented an iron-restricted condition for the purpose of this study.

The Fe content in the duodenum [25] and faeces was measured after digestion in 65% (*w*/*w*) spectrally pure HNO_3_ (Merck) using a microwave digestion system (Speedwave Xpert; Berghof, Eningen, Germany). The Fe levels in the mineral solutions were determined by flame atomic absorption spectrometry (ZA3000; Hitachi, Tokyo, Japan) following digestion and dilution with deionised water. The Fe content in the duodenum and faeces was measured at a wavelength of 248.3 nm. The accuracy of the method was verified using certified reference materials (Bovine Liver 1577C; Sigma-Aldrich, St. Louis, MO, USA) and was determined to be 97%.

Atomic absorption spectrometry (AAS) was used to measure total iron in faecal samples, as overall iron excretion was intended to be measured. AAS is a sensitive, well-established, well standardized, and specific technique broadly employed in pharmacological and nutritional studies to determine total iron content. Its application in iron metabolism research is supported by its high sensitivity, specificity, and minimal sample requirements, which are particularly advantageous when working with small sample volumes such as rat faecal material. Currently, analytical techniques other than AAS require complex and less standardized methodologies. Considering the study’s focus on total iron dynamics, AAS provided a scientifically valid and appropriate approach [42,43].

The HPLA content was determined in duodenal and faecal homogenates using the enzyme-linked immunosorbent assay (ELISA) method. Commercial HPLA ELISA kits validated for small-molecule detection (ELK Biotechnology, Denver, CO, USA) and an Infinite F50 spectrometer (Tecan Group Ltd., Männedorf, Switzerland) were used for the analysis.

### 2.8. Determination of Fe Chelating Ability

The Fe chelating ability was determined using the method described by Decker and Welch, with modifications [44]. A volume of 1 mL of the faeces suspension was added to 2 mM FeCl_2_ (0.05 mL) and 5 mM ferrozine (0.2 mL), a chelating agent. Ferrozine quantitatively forms complexes with Fe^2+^. In the presence of a strong chelator, complex formation is disrupted, leading to a reduction in the red colour intensity of the complex. The degree of colour reduction allows for estimation of the chelating activity of the coexisting chelator. The Fe chelating activity was determined by measuring the reduction in absorbance at 562 nm. Blank samples consisted of phosphate-buffered saline (pH 7.4). The formula for calculating the scavenging ability was [1 − (OD1/OD2)] × 100, where OD1 is the blank absorbance and OD2 is the sample absorbance [45].

The purpose of this analysis was not to directly assess the Fe^3+^ → Fe^2+^ reducing activity of HPLA, but to evaluate the overall Fe^2+^ chelating capacity in faecal samples. The ferrozine-based assay was therefore considered appropriate, as it reflects the ability of the samples to interact with Fe^2+^, the form of iron most relevant for intestinal absorption.

### 2.9. Statistical Analysis

The data are presented as the arithmetic mean ± standard deviation. The normality of the variable distribution was assessed using the Shapiro–Wilk test. Because the data followed a normal distribution, comparisons between groups were conducted using one-way analysis of variance with Tukey’s post hoc test. A *p*-value of <0.05 was considered statistically significant. All statistical analyses were performed using Statistica for Windows 10.0 software (StatSoft, Kraków, Poland). All animals were included in the study analyses.

## 3. Results

No significant differences in **body mass** were observed between the study groups at baseline or at the end of the study [23].

No significant differences in the **HPLA content in the duodenum and faeces** were observed between study groups at the end of the experiment. The HPLA content in the duodenum and faeces is presented in Table 1.

No significant differences in the **Fe chelating ability** in faeces were observed between study groups at the end of the experiment. The Fe chelating ability in faeces is presented in Table 2.

At the end of the experiment, the **duodenal Fe content** was significantly higher in the HFDEFFe group than in the HFDEF group. Additionally, the duodenal Fe content was higher in the HFDEFFeLp and HFDEFFeLc groups than in all other groups except for the HFDEFFe group. The Fe content in the duodenum is presented in Table 3 [25].

The **Fe content in faeces** was significantly higher in the HFDEFFe group than in the C, HF, HFDEF, HFDEFLp, and HFDEFLc groups. Additionally, the Fe content in faeces was significantly higher in the HFDEFFeLp and HFDEFFeLc groups than in the HFDEF and HFDEFLc groups. The Fe content in faeces is presented in Table 4.

The **total faecal bacterial content** was higher in the HFDEFLp group than in the HFDEFFeLc group. Additionally, the total faecal bacterial content at the end of the study was higher in the C group than in the HFDEF, HFDEFFe, and HFDEFFeLc groups. The **lactobacilli content in faeces** was higher in the HFDEFLp and HFDEFLc groups than in the HFDEF, HFDEFFe, and HFDEFFeLc groups. Furthermore, the lactobacilli content in faeces was higher in the C group than in the HF, HFDEF, HFDEFFe, HFDEFFeLp, and HFDEFFeLc groups. The results of the faecal lactobacilli content and total faecal bacterial content are shown in Table 5 [23].

## 4. Discussion

### 4.1. HPLA Content in Duodenum and Faeces and Fe Chelating Ability

Suzuki et al. [18] identified two antioxidants produced by *Lactiplantibacillus plantarum*: HPLA and L-indole-3-lactic acid, with HPLA being the more abundant of the two. Among all the investigated strains of lactobacilli, *Lactiplantibacillus plantarum* was the most effective producer of HPLA, indicating a strong antioxidant effect, which has been confirmed and further investigated [46].

In this study, significant differences in the duodenal HPLA content between the study groups were not observed. Thus, the study did not manage to confirm increased duodenal HPLA production by *Lactiplantibacillus plantarum* in conditions of HF iron-deficient diet. Such observation may indicate, that HPLA synthesis observed previously in vitro [17,18] does not occur in vivo or decreases to a negligible level. The results of this study require further investigation, especially to identify the reasons of diminished HPLA secretion by *L. plantarum* in the gastrointestinal tract. It can be hypothesized that altered conditions in the duodenum or numerous interactions with different microbial species may be the main reason of this.

In our study no statistically significant differences in duodenal HPLA levels were observed among the groups, although some variation in mean values was observed. However, these fluctuations lacked statistical significance. The study successfully detected the presence of HPLA in faeces, but with no significant differences between study groups.

In the study, no significant differences in Fe chelating ability in faeces between the study groups were found. It should be underlined that Fe chelation ability was analysed in the faeces, not in the duodenal content, which allowed to capture chelation processes across the entire gastrointestinal tract rather than just the duodenum or colon. However, the phenomenon of Fe chelation by probiotic bacteria requires further investigation, especially considering the context of this study, as the influence of a HF diet on HPLA production and the Fe chelation abilities of probiotic bacteria remains poorly understood.

Analysing the lack of significant differences in duodenal HPLA content and Fe chelating ability in faeces between the study groups, it should be underlined that there is a range of potential confounding factors, which could have influenced the results of this study. The most important confounding factors are baseline microbiota variability, interaction with other gut metabolites, and the presence of other iron-reducing factors in the gastrointestinal tract [47,48]. Such confounders are unavoidable in in vivo experiments. Despite this limitation of the study, in vivo models much more precisely represent the impact of the investigated intervention on a living organism than the in vitro model.

### 4.2. Fe Content in Duodenum and Faeces

The faecal Fe content was numerically lowest in the HFDEF group, indicating a tendency toward reduced intestinal Fe content under dietary Fe deficiency, although the difference did not reach statistical significance. This observation suggests that the applied HFDEF diet was sufficient to induce at least a partial reduction in available Fe in the intestinal lumen. If probiotics alone intensified intestinal Fe absorption, lower Fe content in faeces in the HFDEFLp and HFDEFLc groups than in the HFDEF group would has been expected, but the results did not support this assumption. This suggests that the ability of *Lactiplantibacillus plantarum* and *Latilactobacillus curvatus* to enhance dietary Fe absorption when supplemented alone is limited. However, the Fe content in faeces tended to be lower in the HFDEFFeLp and HFDEFFeLc groups than in the HFDEFFe group, although this difference did not reach statistical significance. While not conclusive, this trend may indicate a potential for probiotics to modestly support intestinal Fe absorption under dietary Fe deficiency. Though statistically non-significant, such a pattern may suggest a hypothetical synergistic interaction between probiotics and oral iron supplementation. This observation aligns with the duodenal Fe content, which was slightly but insignificantly higher in the HFDEFFeLp and HFDEFFeLc groups than in the HFDEFFe group [25], further suggesting an intensified translocation of orally supplemented Fe from the intestinal content into the duodenum when probiotics were added to Fe supplementation. It is important to note that the colon also has the ability to absorb dietary Fe, with colonic Fe absorption accounting for approximately 10–15% of duodenal absorption [49]. Studies have shown that both DMT1 and ferroportin contribute to colonic Fe absorption [50].

### 4.3. Bacterial Content in Faeces

Higher faecal lactobacilli content in the C group than in the HF and HFDEF groups has been found. As previously mentioned [23], this result clearly indicates that a HF diet disrupts the intestinal microbiota composition by reducing bacterial strains that provide beneficial health effects to the host. The findings of this study align with recent studies showing that a high-fat diet reduces the abundance of beneficial bacteria such as *Lactobacillus*, *Bifidobacterium*, and *Eubacterium*, while increasing enterobacteria and *Bacteroides* [51]. Conversely, some studies suggest that a HF diet, particularly one high in protein, may increase Lactobacillaceae and decrease Clostridiaceae species [52]. Interestingly, in the study, only exclusive supplementation with *Lactiplantibacillus plantarum* or *Latilactobacillus curvatus* successfully restored the faecal lactobacilli content to a level comparable to that of the control group. This suggests that oral probiotic supplementation with these two bacterial strains can counteract the negative effects of a HF diet on intestinal microbiota. Similar conclusions were drawn in a previous study by Moya-Pérez et al. who showed that probiotic supplementation partially reverses gut microbiota alterations induced by an HF diet. These effects were not only observed as a decrease in Firmicutes and lipopolysaccharide-synthesising Proteobacteria but also through reduced synthesis of inflammatory cytokines such as interleukin 6 and 17A, tumour necrosis factor α, and monocyte chemoattractant protein 1, along with a diminished presence of macrophages and B cells. Therefore, the beneficial changes in the gut microbiota resulting from probiotic supplementation are associated with reduced inflammation [53].

The faecal lactobacilli levels were higher in the HFDEFLp and HFDEFLc groups than in the HFDEF and HFDEFFe groups. This suggests that under HF, Fe-deficient dietary conditions *Lactiplantibacillus plantarum* and *Latilactobacillus curvatus*, when administered orally, can survive passage through the gastrointestinal tract and potentially exert their influence within the tract, particularly in the duodenum. This is supported by the observation that the total faecal bacterial content was similar across all four of these groups, further emphasising the potential significance of lactobacilli in faeces for the study’s findings. Interestingly, the faecal lactobacilli content in the HFDEFLp and HFDEFLc groups was statistically comparable to that in HFDEFFeLp but higher than in HFDEFFeLc. This may indicate that under HF diet conditions with Fe supplementation, *Lactiplantibacillus plantarum* was the strain that most effectively survived gastrointestinal passage and was able to exert its influence.

## 5. Study Strengths

In this study, for the first time in an in vivo model, the potential mechanism by which probiotic supplementation could enhance iron absorption beyond traditional pathways was investigated, specifically by examining the possible production of HPLA by *Lactiplantibacillus plantarum* under conditions of a high-fat, iron-deficient diet. Until now, HPLA synthesis by *L. plantarum* had only been demonstrated in in vitro models. Thus, the approach of this study enhanced the physiological relevance of the outcomes. The use of two different bacterial strains, *Lactiplantibacillus plantarum* ATCC 14917 and *Latilactobacillus curvatus* ATCC 25601, allowed for a comparative assessment of strain-specific HPLA production, enhancing the robustness and interpretability of the study findings and providing insights into potential strain-specific probiotic benefits. Additionally, the conditions of a high-fat and iron-deficient diet were investigated, which offered perspective highly relevant to current dietary patterns. In this study, not only the duodenal HPLA content was determined, but also HPLA and Fe content in the faeces and Fe chelation abilities of probiotic strains in the faeces. This multi-faceted approach provided deeper scientific insight into possible effects of probiotic supplementation on Fe.

## 6. Study Limitations

The duration of probiotic supplementation in the study may not fully capture the investigated effects of probiotics under the conditions of the experiment. Therefore, future long-term studies would be beneficial to reveal a broader range of significant intervention effects. Despite the relatively short duration, the study managed to yield some interesting results and trends consistent with results of our previous studies on *Lactiplantibacillus plantarum* ATCC 14917 and *Latilactobacillus curvatus* ATCC 25601 and dietary Fe [23,24,25]. Furthermore, the effects of two probiotic strains at only one dose were investigated, which means that the study does not allow to conclude whether the effects of supplementation are dose-dependent. This aspect requires further investigation. Also, a limitation of the experiment is the use of an ELISA for the determination of duodenal HPLA content. This approach allowed for the efficient analysis of multiple samples and provided valuable comparative data between study groups, however, it may not fully match the specificity and sensitivity of chromatographic techniques such as LC-MS/MS. As potential cross-reactivity with structurally related metabolites cannot be entirely excluded, the findings of the study should be interpreted as indicative of relative changes rather than absolute concentrations. Future studies engaging mass spectrometry–based methods would be valuable to extend the present findings. Another limitation of this study is the relatively high variability observed in some analytical parameters, as reflected by large standard deviations. This variability may be attributed to individual physiological differences among animals, and inherent biological fluctuations. Although such variability may have reduced the statistical power to detect significant between-group differences, the consistent trends observed across parameters still support the overall interpretation of the findings.

## 7. Further Investigations

Probiotic supplementation under conditions of a HF, Fe-deficient diet requires further scientific investigation. The study did not manage to demonstrate that *Lactiplantibacillus plantarum*, despite its ability to synthesize HPLA in vitro, is able to produce increased amounts of this metabolite in the duodenum under conditions of a high-fat, iron-deficient diet in rats. Thus, the findings of the study underscore the importance of validating in vitro probiotic functions in in vivo models before clinical translation. Moreover, future studies should also explore alternative mechanisms by which probiotics may support Fe metabolism.

Specifically, the role of colonic Fe absorption should be clarified because the colon harbours the most abundant bacterial population in the gastrointestinal tract. Because the colon is responsible for approximately 10–15% of Fe absorption compared with the duodenum [49], the Fe content in this organ, along with the synthesis and content of ferroportin and DMT1, should be determined. Additionally, research should focus on which probiotic bacterial strains are most suitable for enhancing dietary Fe absorption in the colon and how this potential increase affects Fe metabolism in the organism under various medical conditions and dietary patterns. Also, future dose–response studies should bring more information on the probiotic dose most optimal for amelioration of dietary iron absorption.

## 8. Clinical Implications

The results of this study, alongside our previous research, can provide a scientific foundation for further experimental and clinical investigations into probiotic supplementation under conditions of Fe deficiency. The findings of the study, together with those of others, could pave the way for the development of interventions based on probiotics to enhance Fe absorption, particularly in patients consuming a high-fat iron-deficient diet. This may contribute to new strategies and guidelines for preventing and managing Fe deficiency, a common and life-threatening nutritional issue worldwide.

## 9. Conclusions

Eight weeks of oral supplementation with *Lactiplantibacillus plantarum* ATCC 14917, whether alone or in combination with oral Fe, does not influence duodenal and faecal HPLA content, nor does it affect faecal Fe chelating abilities in rats on the high-fat, Fe-deficient diet. The influence of probiotic supplementation on Fe metabolism under high-fat, Fe-deficient dietary conditions requires further scientific investigation.

## Figures and Tables

**Table 1:** HPLA content in duodenum and faeces.

Group	*n*	HPLA Content (µg/g)	
		Duodenum	Faeces
**C**	8	27.94 ± 14.05	18.41 ± 2.84
**HF**	8	27.79 ± 16.38	18.50 ± 2.60
**HFDEF**	8	25.90 ± 3.10	19.45 ± 1.36
**HFDEFFe**	8	32.39 ± 16.90	18.61 ± 2.19
**HFDEFLp**	8	31.69 ± 22.33	18.54 ± 2.98
**HFDEFLc**	8	24.74 ± 6.10	20.54 ± 2.20
**HFDEFFeLp**	8	22.90 ± 12.00	18.82 ± 2.21
**HFDEFFeLc**	8	29.68 ± 10.68	21.54 ± 4.07

Data are presented as mean ± standard deviation. HPLA content is presented as HPLA in µg per g of organ and faecal dry mass. Tukey’s honestly significant difference test was implemented.

**Table 2:** Fe chelating ability in faeces.

Group	*n*	Fe Chelating Ability
**C**	8	33.0 ± 16.2
**HF**	8	133.4 ± 97.9
**HFDEF**	8	333.8 ± 151.6
**HFDEFFe**	8	414.5 ± 359.7
**HFDEFLp**	8	72.1 ± 31.4
**HFDEFLc**	8	75.2 ± 53.6
**HFDEFFeLp**	8	378.4 ± 514.7
**HFDEFFeLc**	8	345.9 ± 386.5

Data are presented as mean ± standard deviation. Tukey’s honestly significant difference test was implemented.

**Table 3:** Fe content in duodenum [25].

Group	Duodenal Fe Content (µg/g)
**C**	124.51 ± 50.40 ^acd^
**HF**	141.05 ± 59.69 ^acd^
**HFDEF**	110.49 ± 16.46 ^ac^
**HFDEFFe**	247.06 ± 47.61 ^de^
**HFDEFLp**	170.75 ± 79.84 ^ad^
**HFDEFLc**	183.70 ± 63.42 ^ad^
**HFDEFFeLp**	332.79 ± 108.39 ^be^
**HFDEFFeLc**	293.23 ± 62.85 ^be^

Data are presented as mean ± standard deviation. Mineral content is presented as Fe content in µg per g of organ dry mass. Means not sharing a common superscript (a, b, c, d, e) differ significantly (*p* < 0.05). Tukey’s honestly significant difference test was implemented.

**Table 4:** Fe content in faeces.

Group	Faecal Fe Content (µg/g)
**C**	365.9 ± 105.0 ^ab^
**HF**	251.5 ± 42.4 ^ab^
**HFDEF**	55.1 ± 30.7 ^a^
**HFDEFFe**	752.3 ± 302.6 ^c^
**HFDEFLp**	140.7 ± 33.8 ^ab^
**HFDEFLc**	62.6 ± 22.2 ^a^
**HFDEFFeLp**	431.4 ± 89.1 ^bc^
**HFDEFFeLc**	519.3 ± 254.4 ^bc^

Data are presented as mean ± standard deviation. Mineral content is presented as Fe content in µg per g of faeces dry mass. Means not sharing a common superscript (a, b, c) differ significantly (*p* < 0.05). Tukey’s honestly significant difference test was implemented.

**Table 5:** Faecal microbiological analysis [23].

Group	*n*	Total Faecal Bacterial Content (T)	Lactobacilli Faecal Content (Lb)
**C**	8	9.65 ± 0.24 ^c^	8.49 ± 0.22 ^c^
**HF**	8	8.98 ± 0.08 ^abc^	7.89 ± 0.29 ^ab^
**HFDEF**	8	8.79 ± 0.49 ^ab^	7.47 ± 0.20 ^a^
**HFDEFFe**	8	8.65 ± 0.71 ^ab^	7.54 ± 0.48 ^a^
**HFDEFLp**	8	9.16 ± 0.22 ^bc^	8.16 ± 0.22 ^bc^
**HFDEFLc**	8	9.01 ± 0.49 ^abc^	8.22 ± 0.38 ^bc^
**HFDEFFeLp**	8	8.94 ± 0.87 ^abc^	7.66 ± 0.49 ^ab^
**HFDEFFeLc**	8	8.31 ± 0.55 ^a^	7.57 ± 0.44 ^a^

Data are presented as mean ± standard deviation of log(CFU/g of faeces). CFU—colony forming units. Means not sharing a common superscript (a, b, c) differ significantly (*p* < 0.05). Tukey’s honestly significant difference test was implemented.

## Data Availability

The data supporting the findings of this study are available on reasonable request from the corresponding author.

## Supplementary Materials

The following supporting information can be downloaded at: https://www.mdpi.com/article/10.3390/nu17213454/s1, Table S1: The composition of the animals’ diets in portions with an equal calorific value of 3985 kcal.
