# Supplementary material for: Lactiplantibacillus plantarum, Duodenal Hydroxyphenyllactic Acid and Iron: Insights from a Rat Model of a High-Fat Iron-Deficient Diet
Source: Nutrients. 2025 Nov 1;17(21):3454. doi: 10.3390/nu17213454 (PMC12609434; doi:10.3390/nu17213454)
Supplement: Supplementary file 1 [file nutrients-17-03454-s001.zip › nutrients-3938339-supplementary.pdf]

Supplementary Table S1. The composition of the animals' diets in portions with an equal calorific value of 3985 kcal. [25].

Component	Standard diet [g per 3985 kcal]	High fat diet [g per 3985 kcal]
Corn starch	465.69	152.44
Casein	140.00	140.50
Maltodextrin	155.00	155.00
Saccharose	100.00	100.00
Soybean oil	40.00	25.00
Lard	0.00	154.00
Fiber	50.00	50.00
Mineral mixture (AIN-93m-MX)	35.00	35.00
Vitamin mixture (AIN-93-VX)	10.00	10.00
L-Cystine	1.80	3.00
Choline hydrogen tartrate	2.50	2.50
Tert-butylhydroquinone	0.008	0.008
